# Draft genome sequence of *Stutzerimonas degradans* strain RB, a bacterium capable of complete denitrification

**DOI:** 10.1128/mra.00289-26

**Published:** 2026-06-30

**Authors:** Shujing Yang, Jingjing Wang, Hongyan Wang, Xiuying Li, Huijuan Jin, Jun Yan, Ning Liang, Xiaohong Hou, Yi Yang

**Affiliations:** 1 Institute of Applied Ecology, Chinese Academy of Sciences, Shenyang, Liaoning, China; 2 Shenyang Tianfeng Biological Pharmaceutical Co. Ltd., Shenyang, China; 3 School of Pharmaceutical Engineering, Shenyang Pharmaceutical University 58575, Shenyang, Liaoning, People’s Republic of China; 4 Key Laboratory of Forest Ecology and Silviculture, Institute of Applied Ecology, Chinese Academy of Sciences, Shenyang, Liaoning, China; Montana State University 33052, Bozeman, Montana, USA

**Keywords:** *Stutzerimonas*, denitrification, nitrogen cycle, chlorinated ethenes

## Abstract

*Stutzerimonas degradans* strain RB, capable of complete denitrification, was isolated from earthworm farm sludge. Its draft genome comprises a total length of 4,108,818 bp with 64.4% G + C content, encoding 3,859 protein-coding sequences, 59 tRNAs, and 4 copies each of 5S, 16S, and 23S rRNA genes.

## ANNOUNCEMENT

*Stutzerimonas* is a recently proposed genus within Pseudomonadaceae, comprising strains formerly assigned to the *Pseudomonas stutzeri* group capable of anaerobic growth with nitrate (1, 2). *Stutzerimonas degradans* strains reduce nitrate to N_2_ (3) and degrade contaminants, including chlorinated ethenes (4, 5). To elucidate its denitrification genetics and support nitrogen cycle research, strain RB was isolated in 2017 from a microcosm established with about 2 g of sludge from an earthworm farm in Shenyang, Liaoning Province, China. Isolation used repeated streaking, spread plating, and liquid culture in bicarbonate-buffered mineral salt medium (6) with 5 mM acetate as a carbon source and electron donor and 5 mM nitrate as an electron acceptor under anoxic conditions maintained by N_2_ flushing and bottle sealing. Plates were incubated in an anaerobic glove box. Strain RB reduces nitrate to nitrite and N_2_O, and N_2_O depletion confirms further reduction to N_2_ at 30°C.

Biomass was harvested from a 1.6 L culture with 5 mM nitrate as an electron acceptor. Approximately 5 μg of high-purity genomic DNA was extracted using the HiPure Bacterial DNA Kit (Magen, China) as described (7). Sequencing was performed using PacBio Revio (HiFi) and Illumina NovaSeq Xplus PE150 platforms. For Illumina, a 350 bp insert library was prepared with the NEBNext Ultra DNA Library Prep Kit (New England Biolabs, Ipswich, MA, USA). The library was then sequenced on an Illumina NovaSeq Xplus PE150 instrument (Illumina Inc., USA) with a paired-end approach (2 × 150 bp) and generated 8,301,262 paired-end reads (Q30 = 94.9%). For PacBio, the DNA sample was size selected with a 10 kb BluePippin (Sage Science, Beverly, MA, USA), and the library was prepared using the SMRTbell Template Prep Kit 3.0 (Pacific Biosciences, Menlo Park, CA, USA). The PacBio sequencing generated 41,869 long reads (N_50_ = 13,519 bp). Default settings were used for bioinformatics tools. Raw read quality control, error correction, and adapter trimming were performed using SMRT Link v10.1 (8). The quality of PacBio and Illumina data sets was evaluated using FastQC (9). Raw sequencing reads were assembled using Unicycler v0.50 (10). Completeness and contamination were assessed using CheckM v1.1.3 (11).

The assembled genome of strain RB is represented by four contigs with a total length of 4,108,818 bp (N_50_ = 4,102,969 bp) and a G + C content of 64.5%. Genome completeness and contamination were 100% and 1.14%, respectively. Genome annotation with the NCBI Prokaryotic Genome Annotation Pipeline v6.9 identified a total of 3,859 protein-coding sequences, 59 tRNAs, and 4 copies of 5S, 16S, and 23S rRNA genes (12). GGDC analysis (13) revealed strain RB shares 73.2% dDDH and 97.07% ANI with *Stutzerimonas degradans* DSM 50238 (GCA_024448505), indicating that strain RB belongs to *Stutzerimonas degradans*. Strain RB genome contains all essential genes for complete denitrification, including nitrate reductase (i.e., napE, napA, narJ, narI, and narH), nitrite reductase (i.e., nirS, nirK, nirD, and nirB), nitric oxide reductase (i.e., norV, norR, and norD), and nitrous oxide reductase (i.e., nosD, nosL, and nosZ). This indicates that strain RB possesses a complete denitrification pathway, highlighting its potential for nitrogen cycle regulation.

## Data Availability

Strain RB genome has been deposited in GenBank under accession number GCA_054457595. The BioSample and BioProject numbers are SAMN50587164 and PRJNA1305120, respectively. The raw sequencing reads have been submitted to the Sequence Read Archive under accession numbers SRR35030265 and SRR35030210 for PacBio and Illumina platforms, respectively.
